# Malaria prevalence in HIV-positive children, pregnant women, and adults: a systematic review and meta-analysis

**DOI:** 10.1186/s13071-022-05432-2

**Published:** 2022-09-14

**Authors:** Seyedeh-Tarlan Mirzohreh, Hanieh Safarpour, Abdol Sattar Pagheh, Berit Bangoura, Aleksandra Barac, Ehsan Ahmadpour

**Affiliations:** 1 Student Research Committee, Tabriz University of Medical Sciences, Tabriz, Iran; 2 Immunology Research Center, Tabriz University of Medical Sciences, Tabriz, Iran; 3 Infectious Diseases Research Center, Birjand University of Medical Sciences, Birjand, Iran; 4 Department of Veterinary Sciences, College of Agriculture and Natural Resources, University of Wyoming, Laramie, WY USA; 5 Clinic for Infectious and Tropical Diseases, Clinical Centre of Serbia, 11000 Belgrade, Serbia; 6 Infectious and Tropical Diseases Research Center, Tabriz University of Medical Sciences, Tabriz, Iran; 7 Department of Parasitology and Mycology, Faculty of Medicine, Tabriz University of Medical Sciences, Tabriz, Iran

**Keywords:** AIDS, *Anopheles*, People living with HIV, *Plasmodium*, Protozoan parasite

## Abstract

**Background:**

Malaria in human immunodeficiency virus (HIV)-positive patients is an ever-increasing global burden for human health. The present meta-analysis summarizes published literature on the prevalence of malaria infection in HIV-positive children, pregnant women and adults.

**Methods:**

This study followed the PRISMA guideline. The PubMed, Science Direct, Google Scholar, Scopus and Cochrane databases were searched for relevant entries published between 1 January 1983 and 1 March 2020. All peer-reviewed original papers evaluating the prevalence of malaria among HIV-positive patients were included. Incoherence and heterogeneity between studies were quantified by the I^2^ index and Cochran’s Q test. Publication and population biases were assessed with funnel plots, and Egger’s regression asymmetry test.

**Results:**

A total of 106 studies were included in this systematic review. The average prevalence of malaria among HIV-positive children, HIV-positive pregnant women and HIV-positive adults was 39.4% (95% confidence interval [CI]: 26.6–52.9), 32.3% (95% CI = 26.3–38.6) and 27.3% (95% CI = 20.1–35.1), respectively. In adult patients with HIV, CD4^+^ (cluster of differentiation 4) < 200 cells/µl and age < 40 years were associated with a significant increase in the odds of malaria infection (odds ratio [OR] = 1.5, 95% CI = 1.2–1.7 and OR = 1.1, 95% CI = 1–1.3, respectively). Antiretroviral therapy (ART) and being male were associated with a significant decrease in the chance of malaria infection in HIV-positive adults (OR = 0.8, 95% CI = 0.7–0.9 and OR = 0.2, 95% CI = 0.2–0.3, respectively). In pregnant women with HIV, CD4^+^ count < 200 cells/µl was related to a higher risk for malaria infection (OR = 1.5, 95% CI = 1.1–1.9).

**Conclusions:**

This systematic review demonstrates that malaria infection is concerningly common among HIV-positive children, pregnant women and adults. Among HIV-positive adults, ART medication and being male were associated with a substantial decrease in infection with malaria. For pregnant women, CD4^+^ count of < 200 cells/µl was a considerable risk factor for malaria infection.

**Graphical Abstract:**

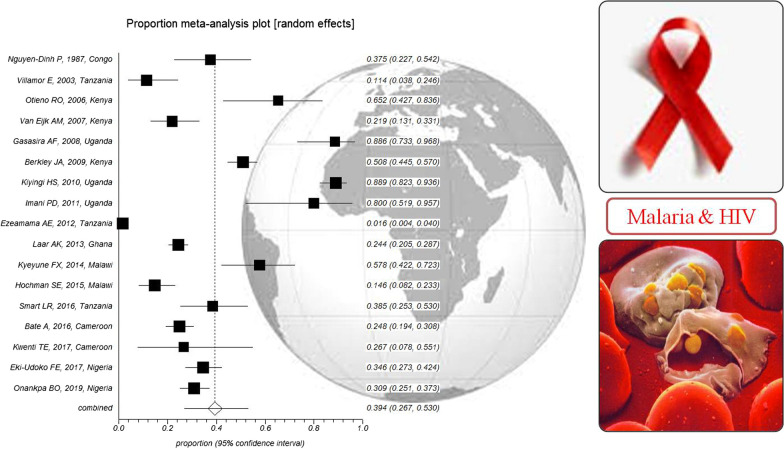

**Supplementary Information:**

The online version contains supplementary material available at 10.1186/s13071-022-05432-2.

## Background

Infectious diseases pose a concerning threat to the health systems of both developed countries and countries with limited resources such as, for example, sub-Saharan countries [1, 2]. With 228 million malaria cases globally in 2018, future declines in the malaria burden caused by *Plasmodium* spp. infections are uncertain [3, 4]. Approximately 3.3 billion people are residing in malaria-endemic regions (parts of the Africa, Southeast Asia and Middle East) [5, 6].

The human immunodeficiency virus (HIV) is an emerging infectious disease agent defined by cellular immune system impairment [7]. HIV is a well-established global health burden, with > 36 million HIV-infected patients and > 1 million HIV-related deaths in 2017 [8]. While *Plasmodium* parasites causing human malaria are transmitted mainly by mosquitoes (*Anopheles* spp.) serving as biological vectors, malaria can also be transmitted directly via blood transfusion, needle sticks with contaminated needles and vertical transmission [9, 10]. The infection routes bypassing the biological vector are transmission routes shared by HIV and malaria [11]. Since HIV infection affects the immune system, the infected individuals are more susceptible to other infections [12–15]. Therefore, people living with HIV (including children, pregnant women and adults) are at risk for significant disease and may have fatal complications following infection [11, 16]. The vertical transmission option for both malaria and HIV facilitates co-transmission from infected pregnant women to their infants [17]. Since the co-infections of malaria and HIV can induce anemia, blood transfusion is often required, but blood transfusion can also contribute to the transmission of HIV and malaria [18, 19].

Although numerous studies have highlighted malaria prevalence in patients with HIV, there has been no comprehensive meta-analysis to demonstrate this prevalence in children, adults and pregnant women. Therefore, the aims of this systematic review and meta-analysis are to summarize malaria prevalence among HIV-positive children, pregnant women and adults, and to identify risk factors that increase the probability of HIV-positive patients being infected with malaria.

## Methods

### Search strategy

For inclusion in the present systematic review, the PubMed, Science Direct, Google Scholar, Scopus and Cochrane databases were searched for relevant English-language, full-text articles and abstracts published between 1 January 1983 (date of HIV discovery) and 1 March 2020. As the aim was to evaluate the prevalence of positive test results for malaria among HIV-positive and HIV-negative individuals, the following Medical Subject Headings (MeSH) terms were used: “Malaria” OR “*Plasmodium*” AND “prevalence” OR “epidemiology” OR “co-infection” AND “HIV” OR “AIDS” OR “acquired immune deficiency syndrome” OR “immunocompromised” OR “immunosuppressed” OR “immunodeficiency” AND “pregnancy women” OR “children” OR “adult” alone OR combined using “OR” and/or “AND”.

### Study selection and data extraction

After an initial search of the databases, subject-related topics and their abstracts were double-checked, and then full texts of potentially eligible articles were selected for downloading. All potentially relevant full texts were reviewed by three independent reviewers (TM, HS, ASP). Discrepancies were resolved by discussion and consensus. The studies were assessed for quality using the Joanna Briggs Institute (JBI) checklist (Additional files 2, 3, 4, 5: Tables S1–S4). The required data were extracted by the reviewers and then re-checked. The criteria for inclusion in the review were: (i) peer-reviewed original research papers; (ii) cross-sectional and cohort studies that estimated the prevalence of malaria infection in HIV-positive and HIV-negative individuals; (iii) published papers in English; (iv) published online before 1 March 2020; and (v) sufficient sample size (*n* > 10). Any article that did not satisfy the above criteria were excluded. The reference lists of the eligible articles were also browsed manually to identify relevant papers that were not initially identified in the database search. Finally, details of each study were extracted using a data extraction form, including country, year of publication, first author, number of HIV^+^ and malaria-positive cases, education status of patients, alcohol consumption status, number of partners, marital status, level of CD4^+^ (cluster of differentiation 4) in HIV-positive patients, ART (antiretroviral therapy) status, sex protection status and diagnostic method (microscopy, serology or molecular). The Preferred Reporting Items for Systematic Reviews and Meta-Analyses (PRISMA) guidelines were used to report the findings [20].

### Meta-analysis

The point estimate and corresponding confidence interval (CI) for the prevalence of malaria in HIV-positive individuals for each study were calculated. Incoherence and heterogeneity among studies were assessed using the I^2^ index and Cochran’s Q test, respectively, and the random-effects model (DerSimonian-Laird) was used for analysis. The heterogeneity among subgroups was tested by meta-regression analysis. The relationship between prevalence, year of publication and sample size was estimated by meta-regression. Additionally, a funnel plot relying on the Egger’s regression asymmetry test was used to assess the small effects of the study and the population bias. For the meta-analysis, the included studies were evaluated as a random sample of each study population, and the analyses were performed using StatsDirect (version 2.7.2) statistical software (StatsDirect Ltd., Altrincham, UK).

## Results

**Fig. 1:**
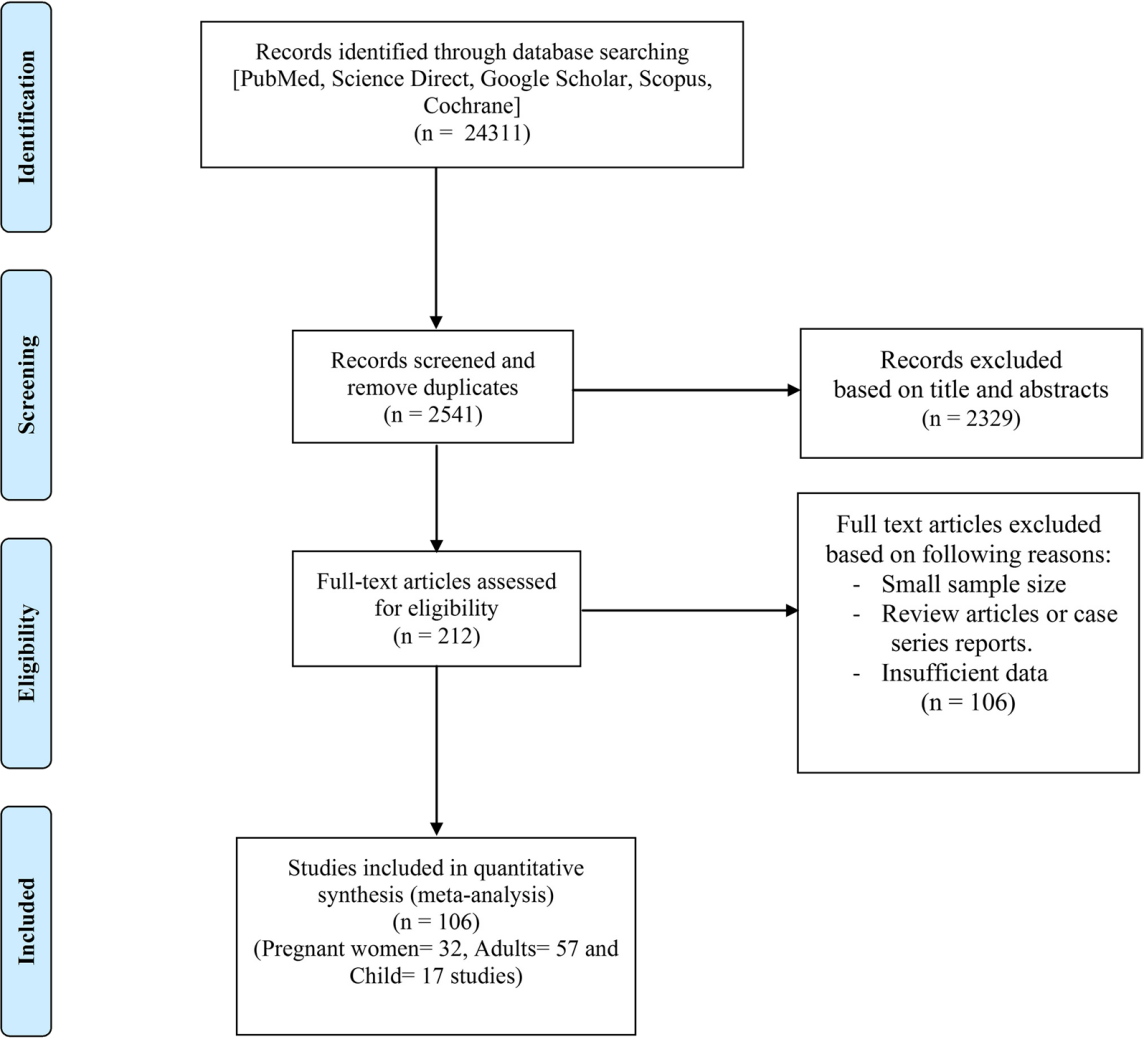
Flowchart of study selection process

The systematic search of the electronic databases identified 24,311 potentially relevant papers. The full-text of 212 articles was assessed, resulting in exclusion from the study of 106 papers due to their small sample size, the review or case report nature of the report, duplication and insufficient data. The remaining 106 papers fulfilled the inclusion criteria and were included in the present systematic review and meta-analysis. All of these 106 articles were published between 1983 and 2020 and present data from malaria-endemic regions in Africa (*n* = 103) and Asia (*n* = 3). The inclusion/exclusion criteria at each step of screening and eligibility and the number of selected papers are shown in Fig. 1.

**Fig. 2:**
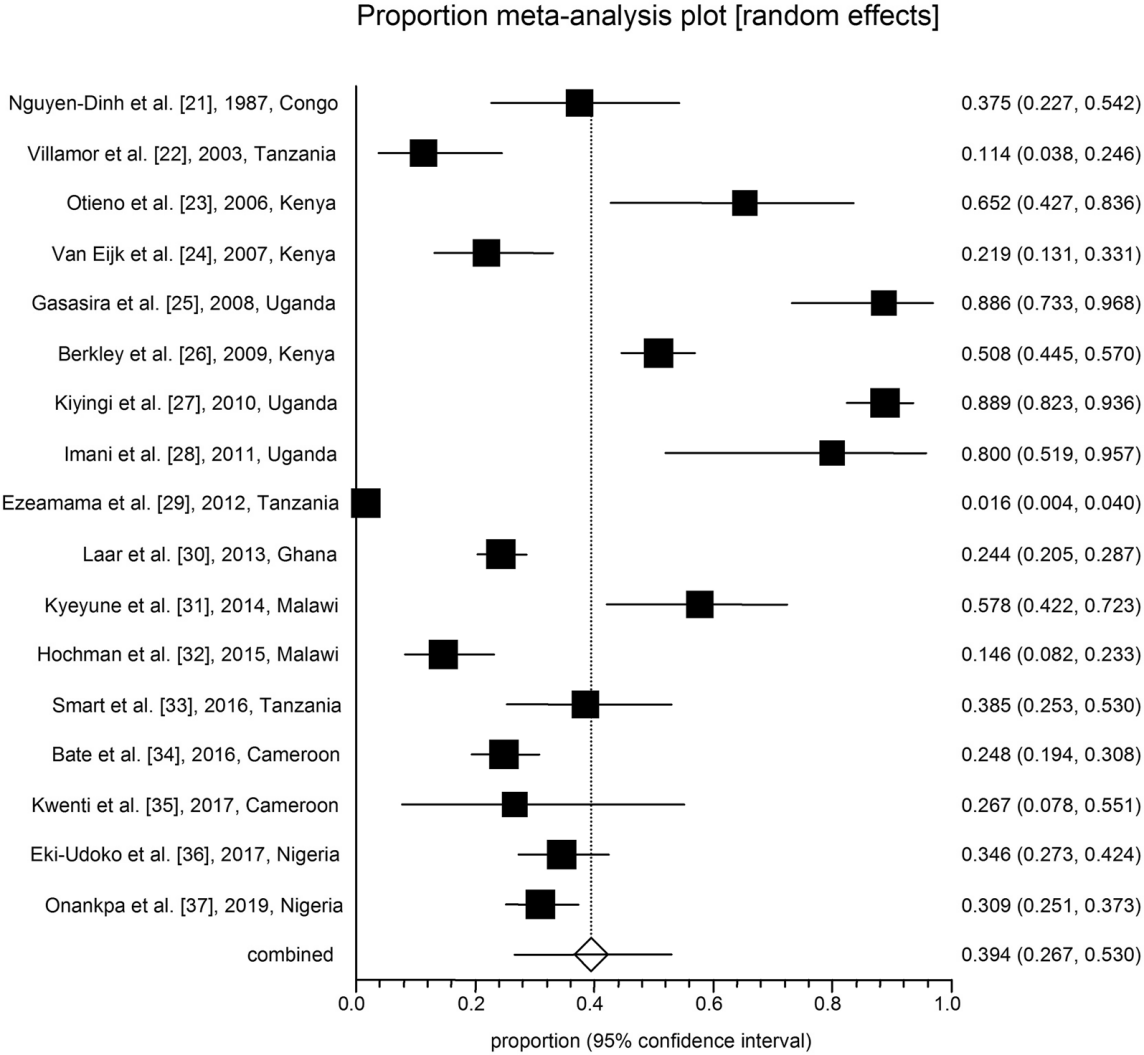
Forest plot diagram of malaria prevalence in human immunodeficiency virus-positive children (first author, year and country)

**Fig. 3:**
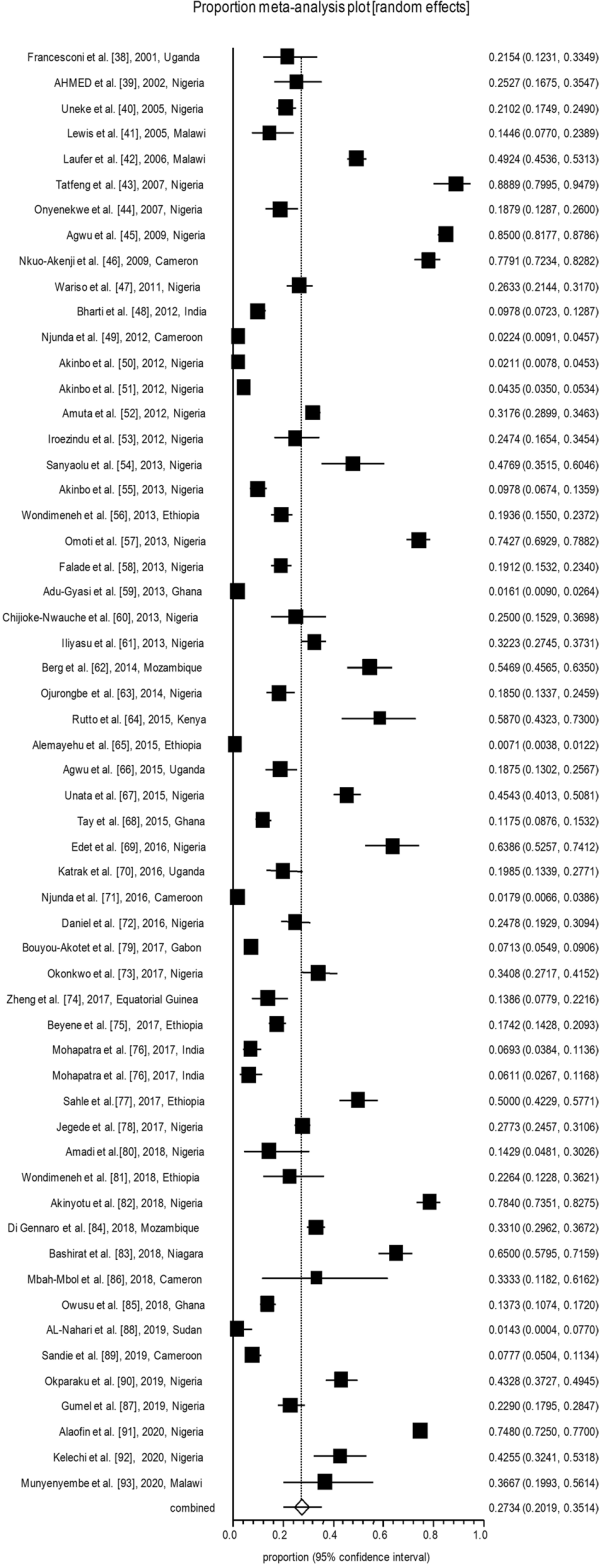
Forest plot diagram of malaria prevalence in human immunodeficiency virus-positive adults (first author, year, and country)

**Fig. 4:**
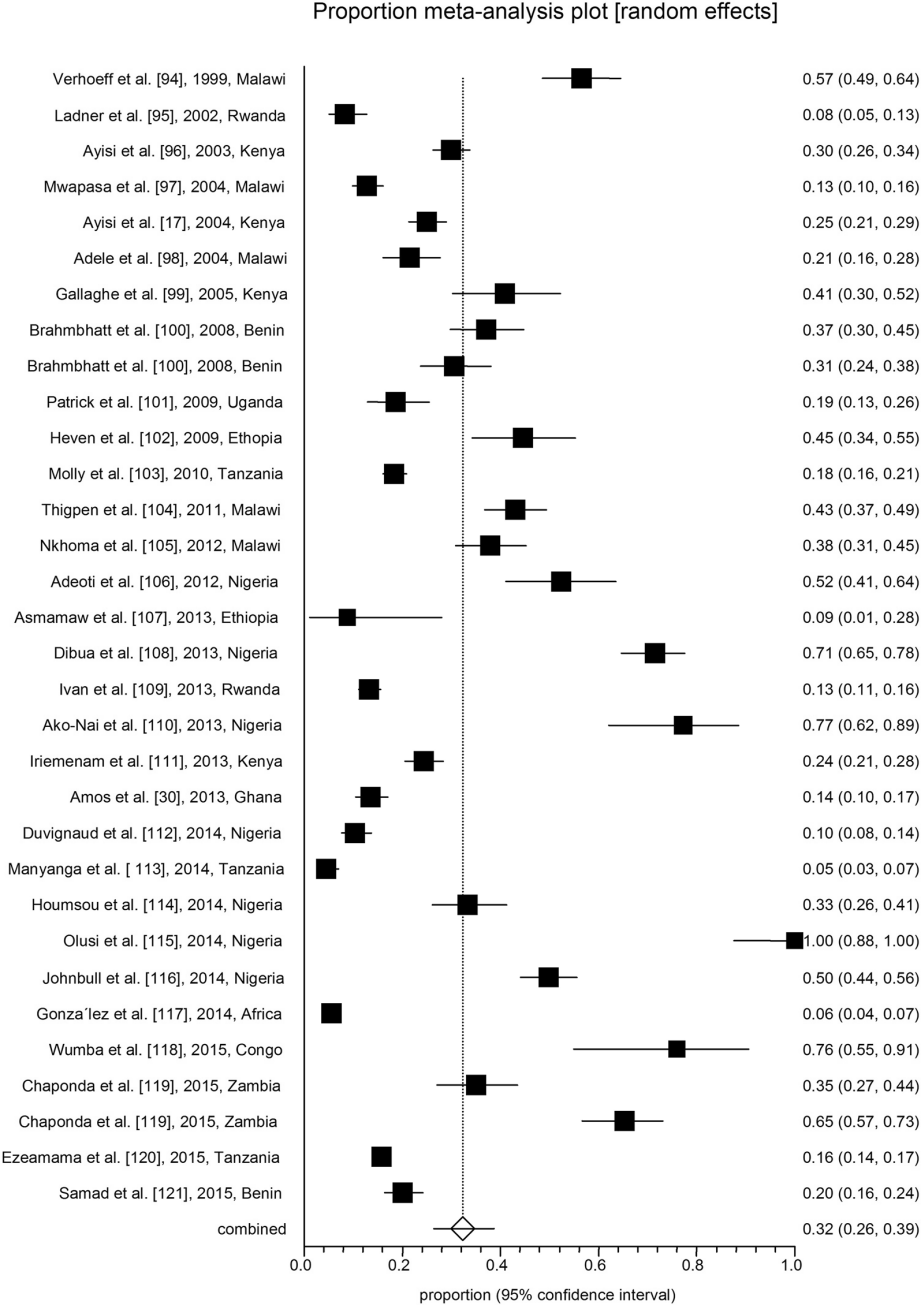
Forest plot diagram of malaria prevalence in human immunodeficiency virus-positive pregnant women (first author, year, and country)

All analyses were conducted in three subgroups: children (*n* = 17; Table 1; Fig. 2), adults (*n* = 57; Table 2; Fig. 3) and pregnant women (*n* = 32; Table 3; Fig. 4). The pooled malaria prevalence among HIV-positive children was 39.4% (95% CI = 26.6–52.9). The combined prevalence of malaria in HIV-positive adults was 27.3% (95% CI = 20.1–35.1), and the collective malaria prevalence among HIV-positive pregnant women was 32.3% (95% CI = 26.3–38.6) (Figs. 2, 3, 4). The funnel plot showing a statistically significant Egger’s regression suggests the possibility of publication bias (Additional file 1: Figure S1). The published risk factors associated with HIV and malaria, namely CD4^+^ level, ART consumption, sex, education, gravidity and age, were analyzed (Table 4). In adult patients with HIV, CD4^+^ count < 200 cells/µl predisposes the patient to malaria infection (odds ratio [OR] = 1.5, 95% CI = 1.2–1.7). In adult HIV-positive patients, age < 40 years old was found to be associated with a significant increase in the odds of being infected with malaria (OR = 1.1, 95% CI = 1–1.3). Also, for adult HIV-positive patients, being male and being treated with ART medication have been associated with a significant decrease in the odds of being infected with malaria (OR = 0.8, 95% CI = 0.7–0.9 and OR = 0.2, 95% CI = 0.2–0.3, respectively). CD4^+^ count < 200 cells/µl was found to predispose pregnant women with HIV to malaria infection (OR = 1.5, 95% CI = 1.1–1.9) (Table 4).

**Table 1:** Baseline characteristics of the included studies on malaria and human immunodeficiency virus co-infection in children

No.	Year of publication	Country/region	Study design	No. of HIV-positive patients	No. of malaria-positive patients	Laboratory diagnostic method	Quality assessment	Reference
1	1987	Zaire (Democratic Republic of Congo)	Case–control	40	15	Blood smear	6/10	[21]
2	2003	Tanzania	Cross-sectional	44	5	Blood smear	6/8	[22]
3	2006	Kenya	Cross-sectional	23	15	Blood smear	7/8	[23]
4	2007	Kenya	Cohort	73	16	Blood smear	8/11	[24]
5	2008	Uganda	Cohort	35	31	Blood smear	8/11	[25]
6	2009	Kenya	Case–control	262	133	Blood smear	8/10	[26]
7	2010	Uganda	Prospective cohort	135	120	Blood smear	8/11	[27]
8	2011	Uganda	Case–control	15	12	Blood smear	9/10	[28]
9	2012	Tanzania	Cohort	255	4	Blood smear	7/11	[29]
10	2013	Ghana	Cross-sectional	443	108	Rapid Test Kit	6/8	[30]
11	2014	Malawi	Cohort	45	26	Blood smear	9/11	[31]
12	2015	Malawi	Cohort	19	15	Autopsy	8/11	[32]
13	2016	Tanzania	Prospective cohort	52	20	Blood smear; rapid diagnostic test; PCR	8/11	[33]
14	2016	Cameroon	Cross-sectional	234	58	Blood smear	8/8	[34]
15	2017	Cameroon	Cross-sectional	15	4	Blood smear	6/8	[35]
16	2017	Nigeria	Cross-sectional	162	56	Blood smear	7/8	[36]
17	2017	Nigeria	Cross-sectional	67	67	Blood smear	5/8	[37]

**Table 2:** Baseline characteristics of the included studies on malaria and human immunodeficiency virus co-infection in adults

No.	Year of publication	Country/region	Study design	No. of HIV-positive patients	No. of malaria-positive patients	Laboratory diagnostic method	Quality assessment	Reference
1	2001	Uganda	Case–control	65	14	Blood smear and ELISA	7/10	[38]
2	2002	Nigeria	Cross-sectional	91	23	Blood smear	6/8	[39]
3	2005	Nigeria	Cross-sectional	490	103	Serology	6/8	[40]
4	2005	Malawi	Cross-sectional	83	12	Blood smear	7/8	[41]
5	2006	Malawi	Cross-sectional	660	325	Blood smear and serology	7/8	[42]
6	2007	Nigeria	Cross-Sectional	81	72	Blood smear	6/8	[43]
7	2007	Nigeria	Prospective study	149	28	RDT	7/11	[44]
8	2008	Cameron	Prospective cohort	258	201	Blood smear	6/11	[45]
9	2009	Nigeria	Cross-sectional	560	476	Blood smear	7/8	[46]
10	2011	Nigeria	Cross-sectional	300	79	RDT	6/8	[47]
11	2012	India	Cohort	460	45	PCR	7/11	[48]
12	2012	Cameroon	Cross-sectional	312	7	Blood smear	8/8	[49]
13	2012	Nigeria	Cross-sectional	285	6	Blood smear	7/8	[50]
14	2012	Nigeria	Cross-sectional	2000	87	Blood smear	7/8	[51]
15	2012	Nigeria	Cross-sectional	1080	343	Blood smear	6/8	[52]
16	2012	Nigeria	Cross-sectional	97	24	Blood smear	8/8	[53]
17	2013	Nigeria	Cross-sectional	65	31	Blood Smear and ELISA	6/8	[54]
18	2013	Nigeria	Cohort	317	31	Blood smear and PCR	7/11	[55]
19	2013	Ethiopia	Retrospective	377	73	Blood smear	9/11	[56]
20	2013	Nigeria	Cross-sectional	342	254	Blood smear	7/8	[57]
21	2013	Nigeria	Cross-sectional	387	74	RDT and blood smear	8/8	[58]
22	2013	Ghana	Cross-sectional	933	15	Blood smear	7/8	[59]
23	2013	Nigeria	Case–control	68	17	Blood smear	8/10	[60]
24	2013	Nigeria	Cross-sectional	363	117	Blood smear	7/8	[61]
25	2014	Mozambique	Cross-Sectional	128	70	Serology and PCR	6/8	[62]
26	2014	Nigeria	Cross-sectional	200	37	PCR	7/8	[63]
27	2015	Kenya	Cross-sectional	46	27	ELISA and blood Smear	7/8	[64]
28	2015	Ethiopia	Cross-Sectional	1819	13	Blood smear and serology	6/8	[65]
29	2015	Uganda	Cross-sectional	160	30	Blood smear	6/8	[66]
30	2015	Nigeria	Cross-sectional	350	159	Blood smear	8/8	[67]
31	2015	Ghana	Cross-sectional	400	47	Blood Smear and serology	7/8	[68]
32	2016	Niagara	Cross-sectional	83	53	Blood smear	7/8	[69]
33	2016	Uganda	Cross-sectional	131	26	LAMP and serology	7/8	[70]
34	2016	Cameroon	Cross-sectional	35	6	Blood smear	7/8	[71]
35	2016	Niagara	Cross-sectional	226	56	Blood smear	6/8	[72]
36	2017	Niagara	Case–control	179	61	PCR and serology	8/10	[73]
37	2017	Equatorial Guinea	Cross-sectional	101	14	Blood smear and ELISA	8/8	[74]
38	2017	Ethiopia	Cross-sectional	528	92	RDT	8/8	[75]
39	2017	India	Prospective cohort	202	14	Blood smear and PCR	8/11	[76]
40	2017	India	Prospective cohort	131	8	Blood smear and PCR	8/11	[76]
41	2017	Ethiopia	Cross-sectional	172	86	Blood smear	7/8	[77]
42	2017	Nigeria	Cross-sectional	761	211	RDT	7/8	[78]
43	2017	Gabon	Cross-sectional	856	61	Blood smear	6/8	[79]
44	2018	Nigeria	Case–control	35	5	PCR and serology	6/8	[80]
45	2018	Ethiopia	Cross-sectional	53	12	Blood smear	7/8	[81]
46	2018	Niagara	Cross-sectional	324	254	Blood smear	7/8	[82]
47	2018	Nigeria	Cross-sectional	200	130	Blood smear	8/8	[83]
48	2018	Mozambique	Retrospective	701	232	RDT	8/11	[84]
49	2018	Ghana	Cross-sectional	466	64	Blood smear	8/8	[85]
50	2018	Cameroon	Cross-sectional	15	5	Blood smear	7/8	[86]
51	2019	Nigeria	Cross-sectional	262	60	Blood smear	8/8	[87]
52	2019	Sudan	Cross-sectional	70	1	PCR	6/8	[88]
53	2019	Cameroon	Cross-sectional	309	24	Blood Smear	8/8	[89]
54	2019	Nigeria	Cross-sectional	268	116	Blood smear	7/8	[90]
55	2020	Niagara	Retrospective	1472	1101	n.a	7/11	[91]
56	2020	Nigeria	Cross sectional	94	40	Serology	8/8	[92]
57	2020	Malawi	Cohort	30	11	Blood smear	8/11	[93]

*ELISA* enzyme-linked immunosorbent assay, *LAMP* loop-mediated isothermal amplification,* n.a.* information not available, *RDT* rapid diagnostic test

**Table 3:** The baseline characteristics of the included studies on malaria and human immunodeficiency virus co-infection in pregnant women

No.	Year of publication	Country/region	Study design	Number of HIV-positive patients	No. of malaria-positive patients	Laboratory diagnostic method	Quality assessment	Reference
1	1999	Malawi	Cross-sectional	159	90	Blood smear	8/8	[94]
2	2002	Rwanda	Cohort	228	19	Blood smear	7/11	[95]
3	2003	Kenya	Cross-sectional	599	179	Blood smear	7/8	[96]
4	2004	Malawi	Cross-sectional	480	61	Blood smear	7/8	[97]
5	2004	Kenya	Cross-sectional	512	128	Blood smear	7/8	[17]
6	2004	Malawi	Cross-sectional	205	44	Blood smear	8/8	[98]
7	2005	Kenya	Cohort	83	34	Smear and/or PCR	7/11	[99]
8	2008	Uganda	Cohort	170	63	IHC	8/11	[100]
9	2008	Uganda	Cohort	170	52	ICT	7/11	[100]
10	2009	Uganda	Cross-sectional	161	30	Blood smear	6/8	[101]
11	2009	Ethiopia	Cross-sectional	92	41	RDT and smear	6/8	[102]
12	2010	Tanzania	Cross-sectional	1006	185	Blood smear	8/8	[103]
13	2011	Malawi	Clinical trial	251	108	Blood smear	11/13	[104]
14	2012	Malawi	Cross-sectional	185	70	Blood smear	8/8	[105]
15	2012	Nigeria	Cross-sectional	82	43	Blood smear	6/8	[106]
16	2013	Ethiopia	Cross-sectional	23	2	Blood smear	7/8	[107]
17	2013	Nigeria	Cohort	203	145	Blood smear	8/10	[108]
18	2013	Rwanda	Cross-sectional	980	130	Blood smear	7/8	[109]
19	2013	Nigeria	Cross-sectional	44	34	Blood smear	7/8	[110]
20	2013	Kenya	Cohort	489	119	Blood smear	8/11	[111]
21	2013	Ghana	Prospective	443	60	RDT	7/11	[30]
22	2014	Nigeria	Cohort	432	45	Smear or RDT	8/11	[112]
23	2014	Tanzania	Cross-sectional	420	19	RDT	8/8	[113]
24	2014	Nigeria	Cross-sectional	159	53	Blood smear	7/8	[114]
25	2014	Nigeria	Cross-sectional	28	28	Blood smear	7/8	[115]
26	2014	Nigeria	Cross-sectional	301	150	Blood smear	6/8	[116]
27	2014	Africa	Randomized controlled trial	973	54	Blood smear	13/13	[117]
28	2015	Congo	Cross-sectional	25	19	Smear and PCR	8/8	[118]
29	2015	Zambia	Cross-sectional	140	49	Blood smear	8/8	[119]
30	2015	Zambia	Cross-sectional	138	90	PCR	7/8	[119]
31	2015	Tanzania	Prospective	2378	376	Clinical	8/11	[120]
32	2015	Benin	Cross-sectional	432	87	Blood smear	7/8	[121]

*ICT* Immunochromatography, *IHC* immunohistochemistry

**Table 4:** Risk factors associated with malaria infection in human immunodeficiency virus-positive patients

Risk factors	Categories	No. study	Odds ratio (95% CI)	*P*-value	*I*^2^ (inconsistency), %	Cochran *Q*	Egger regression test (bias)	*P*-value
*Children*								
ART	Yes No	2	1.3 (0.2–6.6)	0.7342	-	7.3	-	0.0069
CD4+	< 200 cells/µl ≥ 200 cells/µl	2	1.8 (0.8–3.8)	0.1195	-	1.8	-	0.1681
*Adults*								
Sex	Male Female	24	0.8 (0.7–0.9)	0.1393	81.4 (72.9–86.3)	123.4	0.6	0.007
Age (years)	< 40 ≥ 40	20	1.1 (1 -1.3)	0.4716	53 (10.8–70.6)	40.3	0.04	0.0148
ART	Yes No	7	0.2 (0.2–0.3)	0.0029*	82.5 (49.5–90.8)	92.9	1.09	< 0.0001
CD4+	< 200 cells/µl ≥ 200 cells/µl	12	1.5 (1.2–1.7)	0.0428*	90.4 (85.7–93.1)	114.9	1.1	< 0.0001
Education	Primary level Higher-level	3	0.9 (0.7–1.2)	0.8935	0 (0–72.9)	0.5	–	0.9389
*Pregnant women*								
Gravidity	Primigravida Multigravida	9	0.96 (0.7–1.2)	0.9758	38.2 (0–70.2)	12.9	0.2	0.7916
ART	Yes No	4	1.06 (0.7–1.5)	0.96	51.8 (0–82.3)	6.2	0.01	0.1012
CD4+	< 200 cells/µl ≥ 200 cells/µl	4	1.5 (1.1–1.9)	0.7949	92.3 (83.2–95.4)	38.7	− 5.2	0.0012

*ART* Antiretroviral therapy,* CD4* Cluster of differentiation 4,* CI* confidence interval

*Significant association (*P* = 0.05) with malaria infection

## Discussion

Although extensive studies have been conducted on both HIV and *Plasmodium* spp. infections, a comprehensive meta-analysis aimed at precisely evaluating the prevalence of malaria infections among HIV-positive patients and related risk factors is lacking. Therefore, the aim of the present meta-analysis was to provide the pooled prevalence of malaria infection in HIV-positive children, pregnant women and adults and evaluate the related risk factors. The included studies represent African and Asian regions where both HIV and *Plasmodium* spp. are endemic. The pooled malaria prevalence in HIV-positive children, adults and pregnant women included in these studies was 39.4% (95% CI = 26.6–52.9), 27.3% (95% CI = 20.1–35.1) and 32.3% (95% CI = 26.3–38.6), respectively. In adult patients with HIV, receiving ART and having CD4^+^ count > 200 cells/µl were two factors significantly associated with malaria infection (*P* < 0.05).

Due to widespread ART coverage, mortality due to HIV as the main cause of death has decreased drastically over the years [8]. Notwithstanding the extensive efforts to end the acquired immunodeficiency syndrome (AIDS) epidemic by 2030 (set down in the Joint United Nations Program on HIV/AIDS), a lot of the work remains to be done [122]. The troublesome high prevalence of HIV, the increased life expectancy of affected patients, the common co-transmission of HIV and malaria and a remarkable geographical overlap between malaria and HIV high prevalence areas have paved the way for higher rates of co-infections in HIV-positive individuals [123].

Although the incidence of malaria and mortality due to malaria declined significantly by 62% and 41%, respectively, between 2000 and 2015, WHO reported that malaria remained an endemic disease in 76 countries at the beginning of 2016 [124], with approximately 216 million malaria cases in that year. Fifteen countries of the sub-Saharan African region alone were reported to be responsible for 80% of the total malaria burden [125]. Therefore, it is believed that many challenges remain to be overcome in order to eliminate malaria [126]. Regarding the burden of HIV and malaria and the immunosuppressive nature of HIV, there is an urgent need to clarify malaria prevalence in HIV-infected patients and the related risk factors.

According to the results of this systematic review and meta-analysis, the majority of published HIV/malaria studies to date have been in African countries. Socioeconomic conditions and a desirable climate for the biological vector, both of which can facilitate malaria transmission, may be the main reasons underlying this result [127]. Based on our findings, more than one-third of pregnant and HIV-positive women have been infected by malaria, which is worrisome because of the vertical transmission nature of malaria and HIV, which predisposes neonates to other infectious diseases [128, 129]. Indeed, pregnant women are among the most susceptible and vulnerable groups infected with malaria due to the altered immune system during pregnancy [3, 130]. The weakened immune response and HIV infection can lead to even deeper attenuation of the immune system. It is well-recognized that a decline in CD4^+^ cell numbers is associated with attenuation of the cell immune system and an increased vulnerability to being infected with other infections [131]. Our finding that CD4^+^ cell count < 200 cells/µl is linked to increased susceptibility to malaria infection (OR = 1.5, 95% CI = 1.1–1.9) confirms this association. In essence, AIDS and malaria are each controlled by adaptive and innate immune mechanisms, and declining immunity caused by HIV infection will cause an increase in malaria severity. CD4^+^ cells are depleted by the HIV virus, which leads to an impaired immune response to many pathogens, including *Plasmodium* spp. [43]. This pattern was corroborated by Grimwade et al. [132] who observed that malaria incidence in persons with CD4^+^ T cell count ≥ 500/µl, between 200 and 499/μl and < 200/μl was 57, 93 and 140 per 1000 person-year, respectively, in Uganda. It has been postulated that HIV increases malaria incidence in adults based on CD4^+^ cell count categories [133].

This meta-analysis also revealed the worrying situation of malaria infection among HIV-positive children. Approximately 39.4% of HIV-positive children in the analyzed studies were infected with malaria. This is a much higher prevalence than that observed in several studies investigating general children populations in African countries, with the prevalence in these studies ranging from 1% in Kenya to 22% in Uganda, with 14.5% prevalence in Tanzania and 20% in the Democratic Republic of Congo [134, 135]. The observation of increased malaria prevalence in HIV-positive children supports our assumption that susceptibility to co-infection is high in HIV-positive individuals. It is interesting to note that much of the pathogenesis of malaria during pregnancy is mediated by the accumulation of *Plasmodium*-infected red blood cells in the placental intervillous space, termed ‘placental malaria.’ The placenta is also the key interface in mother-to-child transmission of HIV, especially that involving in utero transfer [136]. No remarkable association between receiving ART and HIV infection status has been noted in HIV-positive children (OR = 1.3, 95% CI = 0.2–6.6). Moreover, there has been no significant association between the CD4^+^ cell count and the probability of malaria infection (*P* > 0.05), possibly due to the small number of studies that have considered this factor.

The present meta-analysis reveals that, on average, 27.3% of HIV-positive adults are infected with malaria in endemic countries. One of the consequences of this alarmingly high figure can be manifested in blood transfusion. With the ever-increasing need for a blood transfusion due to environmental and heredity diseases such as sickle cell anemia [137], the prevalence of transfusion-transmitted HIV/malaria can be expected to be high. A study conducted in the sub-African region has demonstrated that about 10–15% of HIV transmission is related to blood transfusion [138]. Ahmadpour et al*.* [19] reported that transfusion-transmitted malaria is a significant challenge in sub-Saharan African regions. In terms of risk factors, CD4^+^ cell count of < 200 cells/µl predisposes HIV-positive adults to *Plasmodium* spp. infection (OR = 1.5, 95% CI = 1.2–1.7). However, the association between malaria and HIV is more complex than expected. Some studies have corroborated that CD4^+^ T cells, as the prime targets for reproduction by HIV-1, play a vital role in immune responses to malaria [131, 139]. Malaria infection leads to upregulation of proinflammatory cytokines and stimulates CD4^+^ cell activation, thus providing the ideal microenvironment for the spread of the HIV virus among the CD4^+^ cells. On the other hand, the selective infection of CD4^+^ cells by HIV leads to the loss of these cells [140]. It is assumed that the increased susceptibility of HIV-seropositive individuals to malaria is related to some immune system-modulating mechanisms, such as depletion of CD4^+^ cells [131, 141].

Age < 40 years has also been associated with a significant increase in the chance of HIV-positive adults becoming infected with malaria (OR = 1.1, 95% CI = 1–1.3). In HIV-positive adults, being male and receiving ART have been associated with a significant decrease in the risk of being infected with *Plasmodium* spp. (OR = 0.8, 95% CI = 0.7–0.9 and OR = 0.2, 95% CI = 0.2–0.3, respectively). This is an interesting finding when compared to individual studies that described a higher risk of malaria infections in males compared to females in the general population in north-east Tanzania, irrespective of their HIV status [134]. Thus, it appears that HIV status may potentially alter malaria susceptibility differently in male patients than in female patients. It is worth emphasizing that the reported figures may not reflect the current status of this co-infection because these endemic areas are limited in terms of healthcare resources, and testing may not be conducted on all people unless they show clinical symptoms. Furthermore, there is insufficient evidence to determine whether or not malaria-induced changes in CD4^+^ T cell counts or viral loads translate to accelerated HIV disease progression or death in areas of stable malaria transmission.

This is the first meta-analysis on malaria prevalence among HIV-positive patients. We broke down the data into three categories, namely infancy, pregnancy and adulthood, and identified the available risk factors for each group. Since there has been little research on the prevalence of malaria in HIV patients in malaria endemic areas, further studies are needed in this regard. Also, due to the incomplete data in the studies included in our meta-analysis, we were unable to evaluate some risk factors, including duration of illness, time of diagnosis and response to treatment. Unfortunately, no data on the health status of individuals having both malaria and HIV infection were provided in these studies. On the other hand, publication bias is one of the main concerns in systematic review studies. As expected, publication bias was observed in the analyzed studies. The main limitation of this systematic review and meta-analysis is related to the different study designs and varying laboratory methods used to determine infection status. Diagnostic methods have varying sensitivity and specificity and, therefore, the heterogeneous prevalence data reported may partially be caused by flaws in methodology. The use of an accurate, reliable and uniform diagnostic techniques would support the correct interpretation of results.

## Conclusions

The current systematic review has revealed concerning prevalence data for malaria among HIV-positive persons, including children, adults and pregnant women. In view of the fact that malaria can quickly become a life-threatening condition in risk groups (e.g. people living with HIV), prevention, chemoprophylaxis, early diagnosis and treatment of clinical malaria are recommended. Recent information also indicates that malaria is associated with the availability of ART and CD4^+^ cell count numbers in adults. Therefore, the related risk factors should be given appropriate attention in HIV/malaria co-infected patients. As HIV infection affects the host immune response, future studies are needed to elucidate the pathogenesis aspects of this co-infection, as well as the severity of its complications, and to investigate possible drugs and drug effectiveness.

## Supplementary Information

**Additional file 1: Figure S1.** Funnel plot of standard error by logit event rate to assess publication or other types of bias across prevalence studies. Studies based on the prevalence of malaria in HIV patients: children (A), adults (B), and pregnant women (C).

**Additional file 2: Table S1.** Summary score for methodological quality of analytic cross-sectional studies.

**Additional file 3: Table S2.** Summary score for methodological quality of analytic case–control studies.

**Additional file 4: Table S3.** Summary score for methodological quality of analytic cohort studies.

**Additional file 5: Table S4.** Summary score for methodological quality of analytic RCT studies.

## Data Availability

The data that support the findings of this study are available from the corresponding author upon reasonable request.

## Abbreviations

AIDS: Acquired immunodeficiency syndrome
ART: Antiretroviral therapy
CD4: Cluster of differentiation 4
HIV: Human immunodeficiency virus
