# Supplementary material for: Malaria prevalence in HIV-positive children, pregnant women, and adults: a systematic review and meta-analysis
Source: Parasit Vectors. 2022 Sep 14;15:324. doi: 10.1186/s13071-022-05432-2 (PMC9472338; doi:10.1186/s13071-022-05432-2)
Supplement: Supplementary file 1 — Additional file 1: Figure S1. Funnel plot of standard error by logit event rate to assess publication or other types of bias across prevalence studies. Studies based on the prevalence of malaria in HIV patients: children (A), adults (B), and pregnant women (C). [file 13071_2022_5432_MOESM1_ESM.doc]

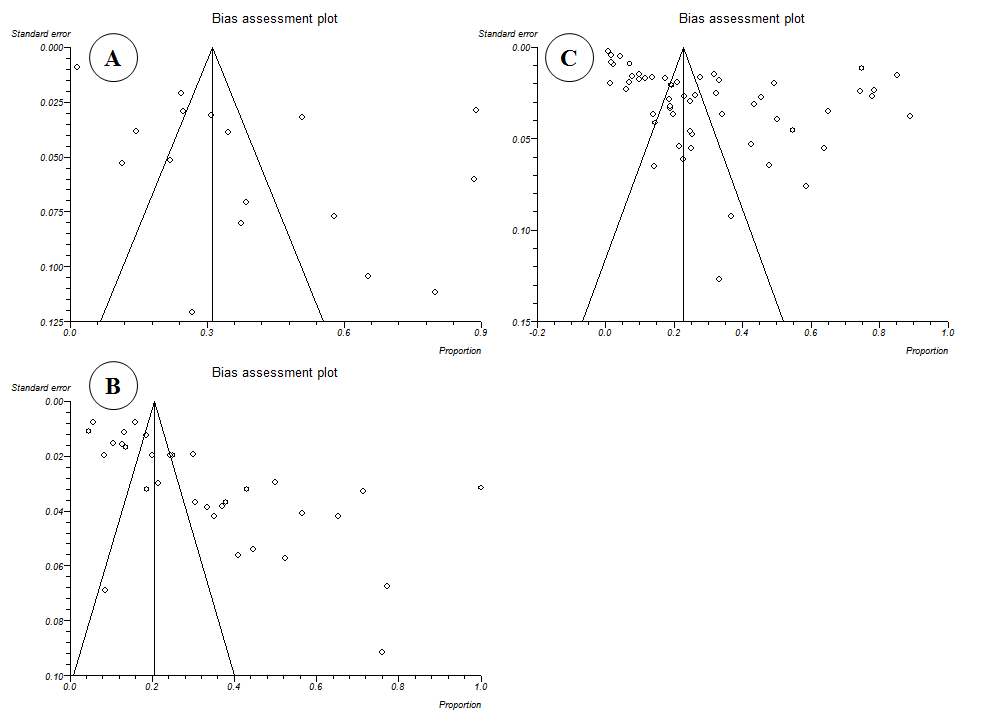


**Figure 1S.** Funnel plot of standard error by logit event rate to assess publication or other types of bias across prevalence studies. Studies based on the prevalence of malaria in HIV patients: Children (A), Adults (B), and Pregnant women (C).
