# Supplementary material for: Malaria prevalence in HIV-positive children, pregnant women, and adults: a systematic review and meta-analysis
Source: Parasit Vectors. 2022 Sep 14;15:324. doi: 10.1186/s13071-022-05432-2 (PMC9472338; doi:10.1186/s13071-022-05432-2)
Supplement: Supplementary file 2 — Additional file 2: Table S1. Summary score for methodological quality of analytic cross-sectional studies. [file 13071_2022_5432_MOESM2_ESM.doc]

**Table 1S.** Summary score for methodological quality of analytic cross-sectional studies

| **Risk assessment of included studies (malaria and HIV co-infection in children)** | | | | | | | | | | |
| --- | --- | --- | --- | --- | --- | --- | --- | --- | --- | --- |
| **ID** | **First author, year of publication** | **Q1** | **Q2** | **Q3** | **Q4** | **Q5** | **Q6** | **Q7** | **Q8** | **Total score** |
| 2 | Villamor E [22], 2003 | U | Y | Y | Y | Y | U | Y | Y | 6/8 |
| 3 | Otieno RO [23], 2006 | U | Y | Y | Y | Y | Y | Y | Y | 7/8 |
| 10 | Laar AK [30], 2013 | Y | Y | Y | Y | U | U | Y | Y | 6/8 |
| 14 | Bate A [34], 2016 | Y | Y | Y | Y | Y | Y | Y | Y | 8/8 |
| 15 | Kwenti TE [35], 2017 | U | Y | Y | Y | Y | U | Y | Y | 6/8 |
| 16 | Eki-Udoko FE [36], 2017 | Y | Y | Y | Y | Y | U | Y | Y | 7/8 |
| 17 | Onankpa BO [37], 2017 | U | U | Y | Y | Y | U | Y | Y | 5/8 |
| **Risk assessment of included studies (malaria and HIV co-infection in adults)** | | | | | | | | | | |
| **ID** | **First author, year of publication** | **Q1** | **Q2** | **Q3** | **Q4** | **Q5** | **Q6** | **Q7** | **Q8** | **Total score** |
| 2 | Ahmed SG [39], 2002 | U | Y | Y | Y | Y | U | Y | Y | 6/8 |
| 3 | Uneke CJ [40], 2005 | Y | U | Y | Y | Y | U | Y | Y | 6/8 |
| 4 | David K. Lewis [41], 2005 | Y | Y | Y | Y | Y | U | Y | Y | 7/8 |
| 5 | Laufer MK [42], 2006 | Y | Y | Y | Y | Y | U | Y | Y | 7/8 |
| 6 | Tatfeng YM [43], 2007 | U | Y | Y | Y | Y | U | Y | Y | 6/8 |
| 9 | Agwu E [46], 2009 | Y | Y | Y | U | Y | Y | Y | Y | 7/8 |
| 10 | Wariso KT [47], 2011 | Y | Y | Y | Y | U | U | Y | Y | 6/8 |
| 12 | Njunda LA [49],2012 | Y | Y | Y | Y | Y | Y | Y | Y | 8/8 |
| 13 | Akinbo FO [50], 2021 | Y | Y | Y | Y | Y | U | Y | Y | 7/8 |
| 14 | Akinbo FO [51], 2012 | Y | Y | Y | Y | Y | U | Y | Y | 7/8 |
| 15 | Amuta EU [52], 2012 | U | Y | Y | Y | Y | Y | U | Y | 6/8 |
| 16 | Michael OI [53], 2012 | Y | Y | Y | Y | Y | Y | Y | Y | 8/8 |
| 17 | Sanyaolu AO [54], 2013 | Y | Y | Y | Y | U | U | Y | Y | 6/8 |
| 20 | Omoti CE [57], 2013 | U | Y | Y | Y | Y | U | Y | Y | 6/8 |
| 21 | Falade CO [58], 2013 | Y | Y | Y | Y | Y | Y | Y | Y | 8/8 |
| 22 | Adu-Gyasi D [59], 2013 | Y | Y | Y | Y | Y | Y | Y | Y | 7/8 |
| 24 | Iliyasu Z [61], 2013 | Y | Y | Y | Y | Y | U | Y | Y | 7/8 |
| 25 | Berg A [62], 2014 | Y | Y | Y | Y | U | U | Y | Y | 6/8 |
| 26 | Ojurongbe O [63], 2014 | Y | Y | Y | Y | Y | U | Y | Y | 7/8 |
| 27 | Rutto EK [64], 2015 | Y | Y | Y | Y | Y | U | Y | Y | 7/8 |
| 28 | Alemayehu G [65], 2015 | Y | Y | Y | Y | U | U | Y | Y | 6/8 |
| 29 | Agwu E [66], 2015 | U | Y | Y | Y | Y | U | Y | Y | 6/8 |
| 30 | Unata IM [67], 2015 | Y | Y | Y | Y | Y | Y | Y | Y | 8/8 |
| 31 | Tay SCK [68], 2015 | Y | Y | Y | Y | Y | U | Y | Y | 7/8 |
| 32 | Edet OU [69], 2016 | Y | Y | Y | Y | Y | Y | Y | Y | 7/8 |
| 33 | Katrak S [70], 2016 | Y | Y | Y | Y | Y | NA | Y | Y | 7/8 |
| 34 | Njunda AL [71], 2016 | Y | Y | Y | Y | Y | U | Y | Y | 7/8 |
| 35 | Daniel, L [72], 2016 | U | Y | Y | Y | Y | NA | Y | Y | 6/8 |
| 37 | Zheng X [74], 2017 | Y | Y | Y | Y | Y | Y | Y | Y | 8/8 |
| 38 | Beyene HB [75], 2017 | Y | Y | Y | Y | Y | Y | Y | Y | 8/8 |
| 41 | Sahle T [77], 2017 | Y | Y | Y | Y | Y | U | Y | Y | 7/8 |
| 42 | Jegede FE [78], 2017 | U | Y | Y | Y | Y | Y | Y | Y | 7/8 |
| 43 | Bouyou Akotet [79], 2017 | Y | Y | Y | Y | U | U | Y | Y | 6/8 |
| 45 | Wondimeneh Y [81], 2018 | Y | Y | Y | Y | Y | U | Y | Y | 7/8 |
| 46 | Akinyotu O [82], 2018 | Y | Y | Y | Y | Y | U | Y | Y | 7/8 |
| 47 | Bello B [83], 2018 | Y | Y | Y | Y | Y | Y | Y | Y | 8/8 |
| 49 | Owusu EDA [85], 2018 | Y | Y | Y | Y | Y | Y | Y | Y | 8/8 |
| 50 | Mbah-Mbol G [86], 2018 | Y | Y | Y | Y | Y | U | Y | Y | 7/8 |
| 51 | Gumel SD [87], 2019 | Y | Y | Y | Y | Y | Y | Y | Y | 8/8 |
| 52 | AL-Nahari W [88], 2019 | Y | Y | Y | Y | U | U | Y | Y | 6/8 |
| 53 | Sandie SM [89], 2019 | Y | Y | Y | Y | Y | Y | Y | Y | 8/8 |
| 54 | Okparaku O [90], 2019 | Y | Y | Y | Y | Y | U | Y | Y | 7/8 |
| 56 | Kelechi C [92], 2020 | Y | Y | Y | Y | Y | Y | Y | Y | 8/8 |
| 57 | Amadi CP [80], 2018 | Y | Y | Y | Y | U | U | Y | Y | 6/8 |
| **Risk assessment of included studies (malaria and HIV co-infection in pregnant women)** | | | | | | | | | | |
| **ID** | **First author, year of publication** | **Q1** | **Q2** | **Q3** | **Q4** | **Q5** | **Q6** | **Q7** | **Q8** | **Total score** |
| 1 | Verhoeff FH [94], 1999 | Y | Y | Y | Y | Y | Y | Y | Y | 8/8 |
| 3 | Ayisi JG [96], 2003 | Y | Y | Y | Y | Y | U | Y | Y | 7/8 |
| 4 | Mwapasa V [97], 2004 | Y | Y | Y | Y | Y | U | Y | Y | 7/8 |
| 5 | Ayisi JG [17], 2004 | U | Y | Y | Y | Y | Y | Y | Y | 7/8 |
| 6 | Adele M Mount [98], 2004 | Y | Y | Y | Y | Y | Y | Y | Y | 8/8 |
| 10 | Newman PM [101], 2009 | U | Y | Y | Y | Y | U | Y | Y | 6/8 |
| 11 | Heven S [102], 2009 | Y | Y | Y | Y | U | U | Y | Y | 6/8 |
| 12 | Franke MF [103], 2010 | Y | Y | Y | Y | Y | Y | Y | Y | 8/8 |
| 14 | Nkhoma ET [105], 2012 | Y | Y | Y | Y | Y | Y | Y | Y | 8/8 |
| 15 | Adeoti OM [106], 2012 | Y | Y | Y | Y | U | U | Y | Y | 6/8 |
| 16 | Asmamaw T [107], 2013 | Y | Y | Y | Y | Y | U | Y | Y | 7/8 |
| 18 | Ivan E [109], 2013 | Y | Y | Y | Y | Y | NA | Y | Y | 7/8 |
| 19 | Ako-Nai KA [110], 2013 | Y | Y | Y | Y | Y | U | Y | Y | 7/8 |
| 23 | Manyanga VP [113], 2014 | Y | Y | Y | Y | Y | Y | Y | Y | 8/8 |
| 24 | Houmsou RS [114], 2014 | Y | Y | Y | Y | Y | U | Y | Y | 7/8 |
| 25 | Olusi TA [115], 2014 | Y | Y | Y | Y | Y | U | Y | Y | 7/8 |
| 26 | Johnbull OS [116], 2014 | Y | Y | Y | Y | U | U | Y | Y | 6/8 |
| 28 | Wumba RD [118], 2015 | Y | Y | Y | Y | Y | Y | Y | Y | 8/8 |
| 29 | Chaponda EB [119], 2015 | Y | Y | Y | Y | Y | Y | Y | Y | 8/8 |
| 30 | Chaponda EB[119], 2015 | Y | Y | Y | Y | Y | U | Y | Y | 7/8 |
| 32 | Samad AI [121], 2015 | Y | Y | Y | Y | Y | U | Y | Y | 7/8 |

**NB: Y = Yes, N = No, U = Unclear, NA = Not Applicable)**

Q1. Were the criteria for inclusion in the sample clearly defined?

Q2. Were the study subjects and the setting described in detail?

Q3. Was the exposure measured in a valid and reliable way?

Q4. Were objective, standard criteria used for measurement of the condition?

Q5. Were confounding factors identified?

Q6. Were strategies to deal with confounding factors stated?

Q7. Were the outcomes measured in a valid and reliable way?

Q8. Was appropriate statistical analysis used?
