# Supplementary material for: Malaria prevalence in HIV-positive children, pregnant women, and adults: a systematic review and meta-analysis
Source: Parasit Vectors. 2022 Sep 14;15:324. doi: 10.1186/s13071-022-05432-2 (PMC9472338; doi:10.1186/s13071-022-05432-2)
Supplement: Supplementary file 3 — Additional file 3: Table S2. Summary score for methodological quality of analytic case–control studies. [file 13071_2022_5432_MOESM3_ESM.doc]

**Table 2S.** Summary score for methodological quality of analytic case-control studies

| **Risk assessment of included studies (malaria and HIV co-infection in children)** | | | | | | | | | | | | |
| --- | --- | --- | --- | --- | --- | --- | --- | --- | --- | --- | --- | --- |
| ID | **First author, year of publication** | **Q1** | **Q2** | **Q3** | **Q4** | **Q5** | **Q6** | **Q7** | **Q8** | **Q9** | **Q10** | **Total score** |
| 1 | Nguyen-Dinh P [21], 1987 | NA | Y | U | Y | Y | Y | U | Y | NA | Y | 6/10 |
| 6 | Berkley JA [26], 2009 | Y | Y | Y | Y | Y | Y | U | Y | NA | Y | 8/10 |
| 8 | Imani PD [28], 2011 | Y | Y | Y | Y | Y | Y | Y | Y | U | Y | 9/10 |
| **Risk assessment of included studies (malaria and HIV co-infection in adults)** | | | | | | | | | | | | |
| ID | **First author, year of publication** | **Q1** | **Q2** | **Q3** | **Q4** | **Q5** | **Q6** | **Q7** | **Q8** | **Q9** | **Q10** | **Total score** |
| 1 | Francesconi P [38], 2001 | U | Y | Y | Y | Y | Y | U | Y | U | Y | 7/10 |
| 23 | Chijioke-Nwauche I [60], 2013 | Y | Y | Y | Y | Y | Y | U | Y | U | Y | 8/10 |
| 36 | Okonkwo I [73], 2017 | Y | Y | Y | Y | Y | Y | U | Y | U | Y | 8/10 |
| **Risk assessment of included studies (malaria and HIV co-infection in pregnant women)** | | | | | | | | | | | | |
| ID | **First author, year of publication** | **Q1** | **Q2** | **Q3** | **Q4** | **Q5** | **Q6** | **Q7** | **Q8** | **Q9** | **Q10** | **Total score** |
| 17 | Uju MD [108], 2013 | Y | Y | Y | Y | Y | Y | NA | Y | U | Y | 8/10 |

**(NB: Y = Yes, N = No, U = Unclear, NA = Not Applicable)**

Q1. Were the groups comparable other than the presence of disease in cases or the absence of disease in controls?

Q2. Were cases and controls matched appropriately?

Q3. Were the same criteria used for identification of cases and controls?

Q4. Was exposure measured in a standard, valid and reliable way?

Q5. Was exposure measured in the same way for cases and controls?

Q6. Were confounding factors identified?

Q7. Were strategies to deal with confounding factors stated?

Q8. Were outcomes assessed in a standard, valid and reliable way for cases and controls?

Q9. Was the exposure period of interest long enough to be meaningful?

Q10. Was appropriate statistical analysis used?
