# Supplementary material for: Malaria prevalence in HIV-positive children, pregnant women, and adults: a systematic review and meta-analysis
Source: Parasit Vectors. 2022 Sep 14;15:324. doi: 10.1186/s13071-022-05432-2 (PMC9472338; doi:10.1186/s13071-022-05432-2)
Supplement: Supplementary file 4 — Additional file 4: Table S3. Summary score for methodological quality of analytic cohort studies. [file 13071_2022_5432_MOESM4_ESM.doc]

**Table 3S.** Summary score for methodological quality of analytic cohort studies

| **Risk of assessment of included studies (malaria and HIV co-infection in children)** | | | | | | | | | | | | | |
| --- | --- | --- | --- | --- | --- | --- | --- | --- | --- | --- | --- | --- | --- |
| **ID** | **First author, year of publication** | **Q1** | **Q2** | **Q3** | **Q4** | **Q5** | **Q6** | **Q7** | **Q8** | **Q9** | **Q10** | **Q11** | **Total Score** |
| **4** | Van Eijk AM [24], 2007 | Y | Y | Y | Y | U | Y | Y | Y | U | NA | Y | 8/11 |
| **5** | Gasasira AF [25], 2008 | Y | Y | Y | Y | U | Y | Y | U | Y | NA | Y | 8/11 |
| **7** | Kiyingi HS [27], 2010 | Y | Y | Y | Y | U | Y | Y | Y | U | U | Y | 8/11 |
| **9** | Ezeamama AE [29], 2012 | Y | Y | Y | U | U | Y | Y | U | Y | NA | Y | 7/11 |
| **11** | Kyeyune FX [31], 2014 | Y | Y | Y | U | NA | Y | Y | Y | Y | Y | Y | 9/11 |
| **12** | Hochman SE [32], 2015 | Y | Y | Y | U | U | Y | Y | U | Y | Y | Y | 8/11 |
| **13** | Smart LR [33], 2016 | Y | Y | Y | U | NA | Y | Y | Y | U | Y | Y | 8/11 |
| **Risk of assessment of included studies (malaria and HIV co-infection in adults)** | | | | | | | | | | | | | |
| **ID** | **First author, year of publication** | **Q1** | **Q2** | **Q3** | **Q4** | **Q5** | **Q6** | **Q7** | **Q8** | **Q9** | **Q10** | **Q11** | **Total Score** |
| **7** | Onyenekwe CC [44], 2007 | Y | Y | Y | U | U | Y | Y | Y | NA | NA | Y | 7/11 |
| **8** | Nkuo-Akenji [45], 2008 | Y | Y | Y | U | U | Y | Y | U | U | NA | Y | 6/11 |
| **11** | Bharti AR [48], 2012 | Y | Y | Y | U | U | Y | Y | Y | U | U | Y | 7/11 |
| **18** | Akinbo FO [55], 2013 | Y | Y | Y | U | U | Y | Y | U | Y | U | Y | 7/11 |
| **19** | Wondimeneh Y [56], 2013 | Y | Y | Y | U | U | Y | Y | Y | Y | Y | Y | 9/11 |
| **39** | Mohapatra PK [76], 2017 | Y | Y | Y | Y | NA | Y | Y | Y | U | NA | Y | 8/11 |
| **40** | Mohapatra PK [76], 2017 | Y | Y | Y | Y | U | Y | Y | Y | U | NA | Y | 8/11 |
| **48** | Di Gennaro F [84], 2018 | Y | Y | Y | Y | U | Y | Y | Y | U | U | Y | 8/11 |
| **55** | Alaofin OS [91], 2020 | Y | Y | Y | Y | U | Y | Y | U | U | NA | Y | 7/11 |
| **57** | Munyenyembe AU [93], 2020 | Y | Y | Y | Y | U | Y | Y | Y | U | NA | Y | 8/11 |
| **Risk of assessment of included studies (malaria and HIV co-infection in pregnant women)** | | | | | | | | | | | | | |
| **ID** | **First author, year of publication** | **Q1** | **Q2** | **Q3** | **Q4** | **Q5** | **Q6** | **Q7** | **Q8** | **Q9** | **Q10** | **Q11** | **Total Score** |
| **2** | Ladner J [95], 2002 | Y | Y | Y | U | NA | Y | Y | Y | U | U | Y | 7/11 |
| **7** | Gallagher M [99], 2005 | Y | Y | Y | Y | U | Y | Y | U | U | U | Y | 7/11 |
| **8** | Brahmbhatt H [100], 2008 | Y | Y | Y | Y | U | Y | Y | Y | U | NA | Y | 8/11 |
| **9** | Brahmbhatt H [100], 2008 | Y | Y | Y | U | U | Y | Y | U | Y | NA | Y | 7/11 |
| **20** | Iriemenam NC [111], 2013 | Y | Y | Y | Y | U | Y | Y | Y | U | NA | Y | 8/11 |
| **21** | Amos K Laar [30], 2013 | Y | Y | Y | U | U | Y | Y | Y | U | U | Y | 7/11 |
| **22** | Duvignaud A [112], 2014 | Y | Y | Y | Y | U | Y | Y | Y | U | U | Y | 8/11 |
| **31** | Samad AI [121], 2015 | Y | Y | Y | Y | U | Y | Y | U | Y | NA | Y | 8/11 |

**(NB: Y = Yes, N = No, U = Unclear, NA = Not Applicable)**

Q1. Were the two groups similar and recruited from the same population?

Q2. Were the exposures measured similarly to assign people to both exposed and unexposed groups?

Q3. Was the exposure measured in a valid and reliable way?

Q4. Were confounding factors identified?

Q5. Were strategies to deal with confounding factors stated?

Q6. Were the groups/participants free of the outcome at the start of the study (or at the moment of exposure)?

Q7. Were the outcomes measured in a valid and reliable way?

Q8. Was the follow up time reported and sufficient to be long enough for outcomes to occur?

Q9. Was follow up complete, and if not, were the reasons to loss to follow up described and explored?

Q10. Were strategies to address incomplete follow up utilized?

Q11. Was appropriate statistical analysis used?
