# Supplementary material for: Malaria prevalence in HIV-positive children, pregnant women, and adults: a systematic review and meta-analysis
Source: Parasit Vectors. 2022 Sep 14;15:324. doi: 10.1186/s13071-022-05432-2 (PMC9472338; doi:10.1186/s13071-022-05432-2)
Supplement: Supplementary file 5 — Additional file 5: Table S4. Summary score for methodological quality of analytic RCT studies. [file 13071_2022_5432_MOESM5_ESM.doc]

**Table 4S.** Summary score for methodological quality of analytic RCT studies

| **Risk of assessment of included studies (malaria and HIV co-infection in pregnant women)** | | | | | | | | | | | | | | | |
| --- | --- | --- | --- | --- | --- | --- | --- | --- | --- | --- | --- | --- | --- | --- | --- |
| **ID** | **First author, year of publication** | **Q1** | **Q2** | **Q3** | **Q4** | **Q5** | **Q6** | **Q7** | **Q8** | **Q9** | **Q10** | **Q11** | **Q12** | **Q13** | **Total score** |
| **13** | Thigpen MC [104], 2011 | Y | Y | Y | Y | U | U | Y | Y | Y | Y | Y | Y | Y | 11/13 |
| **27** | Gonza´lez R [117], 2014 | Y | Y | Y | Y | Y | Y | Y | Y | Y | Y | Y | Y | Y | 13/13 |

**(NB: Y = Yes, N = No, U = Unclear, NA = Not Applicable)**

Q1. Was true randomization used for assignment of participants to treatment groups?

Q2. Was allocation to treatment groups concealed?

Q3. Were treatment groups similar at the baseline?

Q4. Were participants blind to treatment assignment?

Q5. Were those delivering treatment blind to treatment assignment?

Q6. Were outcomes assessors blind to treatment assignment?

Q7. Were treatment groups treated identically other than the intervention of interest?

Q8. Was follow up complete and if not, were differences between groups in terms of their follow up adequately described and analyzed?

Q9. Were participants analyzed in the groups to which they were randomized?

Q10. Were outcomes measured in the same way for treatment groups?

Q11. Were outcomes measured in a reliable way?

Q12. Was appropriate statistical analysis used?

Q13. Was the trial design appropriate, and any deviations from the standard RCT design (individual randomization, parallel groups) accounted for in the conduct and analysis of the trial?
